# Eight-week virtual reality training improves lower extremity muscle strength but not balance in adolescents with intellectual disability: A randomized controlled trial

**DOI:** 10.3389/fphys.2022.1053065

**Published:** 2022-11-22

**Authors:** Shuhan Wang, Hao Yu, Zhaoxia Lu, Jiangna Wang

**Affiliations:** Shandong Sport University, Jinan, China

**Keywords:** virtual reality, intellectual disability, balance, muscle strength, adolescents

## Abstract

**Purpose:** This study aims to assess the effects of 8-week virtual reality (VR) training on balance and lower extremity muscle strength in adolescents with intellectual disability (ID).

**Methods:** Thirty adolescents with intellectual disability were randomly divided into the virtual reality group and control group. The participants in the virtual reality group and the control group received the virtual reality training and the physical education (PE) course, respectively, for 8 weeks. The Berg Balance Scale (BBS), Timed Up and Go (TUG) test and lower extremity muscle strength were measured before and after the training.

**Results:** The between-group results showed that the participants in the virtual reality group increased the muscle strength of hip flexors (*p* < 0.001), hip extensors (*p* = 0.002), hip abductors (*p* < 0.001), knee flexors (*p* < 0.001), knee extensors (*p* = 0.002) and ankle plantar flexors (*p* = 0.042) significantly after training, compared to the control group. However, no significant improvement was found in the berg balance scale and timed up and go scores between the virtual reality group and control group after training (*p* > 0.05). The within-group results showed that the strength of all the muscle groups significantly increased after training in the virtual reality group (*p* < 0.05) compared to the baseline. However, no significant difference was found in the muscle strength in the control group before and after training. The within-group berg balance scale and timed up and go scores showed no significant improvements in both groups.

**Conclusion:** Virtual reality training intervention might be effective in improving the lower extremity muscle strength, but no significant improvement was found on balance ability in adolescents with intellectual disability.

## 1 Introduction

Intellectual disability (ID) is a developmental disorder characterized by limitations on intellectual functioning and adaptive behaviour. These limitations arise before the age of 22 (Schalock et al., 2021). The prevalence of ID is approximately 1% (McKenzie et al., 2016). This disability may affect the nervous and/or sensory system and cause functional and physical disability (Manel Font-Farré et al., 2021). Previous studies showed that high level of physical fitness during adolescence could be a predictor of high level of physical activity in adulthood (Pertti Huotari et al., 2011). Some studies suggest that individuals with ID have a low level of physical activities compared with their typically developing peers. This might indicate that the physical fitness in ID adolescents is relatively low since they have a low level of physical activities. In addition, low level of physical fitness, including poor muscular strength and balance may result in impaired stability and increase risk of falls (Westendorp et al., 2011; Blomqvist et al., 2013; Einarsson et al., 2015; Hocking et al., 2016; Hsu., 2016; Wouters et al., 2020). Individuals with ID are prone to falls and injuries (Enkelaar et al., 2012). A review paper reported that the proportion of people with ID who were subjected to falls was 39% [95% CI (0.35–0.43)](Ho et al., 2019). For example, individuals with ID are more likely to have fall-related fractures due to low bone mineral density. Compared with the healthy population, the rate of hospitalization due to injuries was as twice as high in the ID population, and most of these injuries were fall-related (Enkelaar et al., 2012). Poor balance control might be related to the falls in the ID population. (Hsu, 2016). Balance control is closely related to lower extremity muscle strength (J C Nitz et al., 2010). Good muscle strength and balance control are crucial for preventing falls in physical activities of daily life, such as walking and stair climbing (Muehlbauer et al., 2015; Obrusnikova et al., 2021). Therefore, improving muscle strength and balance control is necessary for individuals with ID.

Different trainings can have positive effects on motor function; however, they also have limitations. Several physical therapies have been used to improve muscle strength and balance in individuals with ID, including resistance training (Cowley et al., 2011; Obrusnikova et al., 2021), sensorimotor gymnastics (Jankowicz-Szymanska et al., 2012), balance training (Lee et al., 2016), combined training program (Kachouri et al., 2016), physical education (PE) (Gupta et al., 2011) and others. Obrusnikova et al. and Cowley reported that resistance training could significantly increase the muscular strength of individuals with ID (Cowley et al., 2011; Obrusnikova et al., 2021). Jankowicz-Szymanska found that systematic sensorimotor gymnastics can improve static balance ability in young people with ID (Jankowicz-Szymanska et al., 2012). Lee et al. found that balance training had the effects on improving the postural balance in adolescents with ID (Lee et al., 2016). In addition, Kachouri et al. suggested a training program combined strength and proprioceptive training had the effects on improving the muscle strength and postural balance in children with ID (Kachouri et al., 2016). However, the review paper (Jeng et al., 2015) showed that exercise training is ineffective in improving balance and coordination in adolescents with ID. The effects of physical training on balance and muscle strength are inconsistent. In addition, PE is an important opportunity to promote exercise in adolescents with ID. PE is widely used as a control condition in previous studies, which aimed to investigate the effects of specific training programs in improving the muscle functions and balance (Gupta et al., 2011). For example, Gupta et al. found that regular physical activities followed at school may improve the strength and balance in children with ID (Gupta et al., 2011). And another study also showed PE can improve lower extremity muscle strength in students with ID (Hsu., 2016). However, evidence for isolated or limited social interactions and less physical engagement during PE course were found in students with ID compared to their healthy peers (Erica Gobbi et al., 2018). Less social interactions and physical engagements may be attributed to the lack of enjoyment for adolescents with ID during PE course. Consequently, an attractive and effective physical training is necessary to improve the balance and muscle strength for individuals with ID.

Virtual reality (VR) plays an increasingly important role in motor rehabilitation (Juras et al., 2019). VR is defined as “the use of interactive simulations created by computer to provide users with environments” (Weiss et al., 2006). The use of interactive video games as a form of motor rehabilitation can improve motivation, exercise performance, and tolerance in children (Ferguson et al., 2013). It stimulates the brain in a multisensory manner by creating temporary interaction scenarios that involve all the senses, and adds motivation and pleasure in a game-like environment, which creates good training experiences (Leeb and Perez-Marcos, 2020). The convergence of VR gaming with exercise have shown good results in physical, mental and social health (Nathan Keller et al., 2022). Therefore, VR training may have better training compliance compared to traditional physical trainings methods. Previous study showed that a minimum of 8 weeks of VR training should be done for the training effect to show up (Lee et al., 2019). Few studies showed the effect of VR training on balance and lower extremity muscle strength in adolescents with ID. In previous studies, VR has been used to improve the functional abilities of individuals with ID (Patrice L Tamar Weiss et al., 2003; Shira Yalon-Chamovitz and Weiss, 2008; Lotan et al., 2009; Ahn, 2021). Shira et al. showed that VR appeared to offer diverse and stimulating experiences during leisure activities among individuals with ID. In addition, VR can be easily combined with physical training, which is highly adaptable and suitable for the ID population (Shira Yalon-Chamovitz and Weiss, 2008). Lotan et al. found that VR training had the effects on improving the physical fitness of individuals with ID (Lotan et al., 2009). Ahn showed that VR and computer game-based cognitive therapy for visual-motor integration is an effective training method for children with ID to promote visual perception and motor function (Ahn, 2021). Furthermore, some studies have shown that VR training can improve the balance and muscle strength of older adults and individuals with neurological diseases, such as patients suffering from stroke, Parkinson’s disease and cerebral palsy (Cho et al., 2016; Lee et al., 2019; Mak and Wong-Yu, 2019; Orsatti-Sánchez. and Diaz-Hernandez., 2021; Sadeghi et al., 2021).

The present study aimed to investigate the effects of VR training on balance and lower extremity muscle strength in adolescents with ID. We hypothesized that b: 1) The participants in VR group and PE group would have significant improvement on balance and lower extremity muscle strength after training. 2) The VR training could be more efficient than PE course for improving balance and lower extremity muscle strength in adolescents with ID.

## 2 Materials and methods

### 2.1 Study design

**FIGURE 1:**
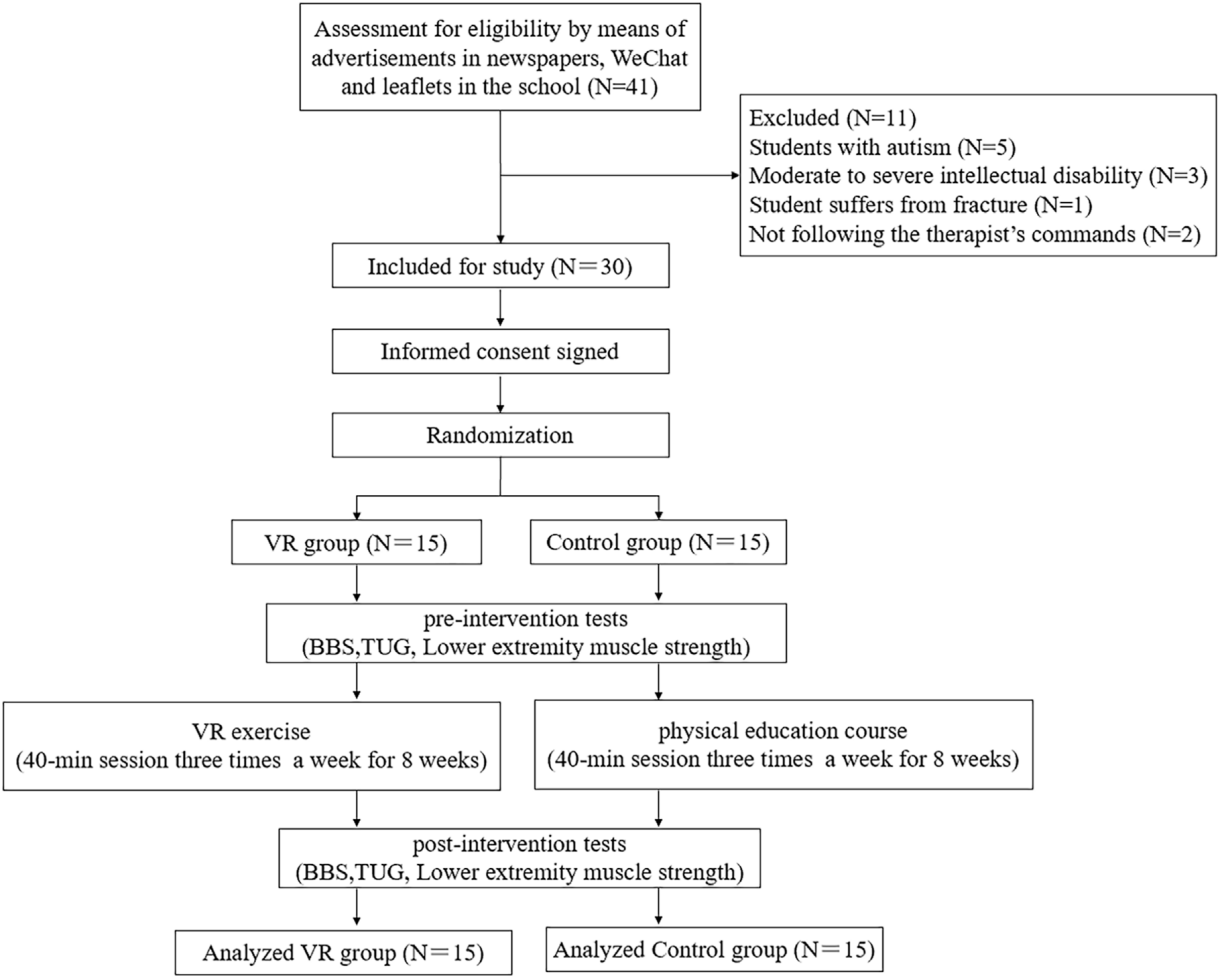
Flow diagram for randomized controlled trial.

A randomized controlled trial was designed to evaluate the effect of 8-week VR training on balance and lower extremity muscle strength (Figure 1). The participants in VR group and control group received the VR training and PE course for 8 weeks, respectively. Muscle strength and balance control were measured at the baseline and immediately after training.

### 2.2 Participants

#### 2.2.1 Sample size estimation

G*Power software (Version 3.1.9.2) was used to estimate the sample size. The following data were determined: effect size = 0.57 (Hsu., 2016), two-tailed significance, statistical power = 0.8, *α* = 0.05. Accounting for a drop-out rate of 20% (Wang et al., 2021), 15 participants were recruited per group in this study.

#### 2.2.2 Participant recruitment and randomization

In this study, 41 adolescents were contacted from a special education school. A total of 30 participants (male = 11, female = 19) aged 11–18 years old who were diagnosed with ID by means of advertisements in newspapers, WeChat and leaflets in the school met the study eligibility criteria and participated in this study. All the participants were randomly assigned to the VR group (*n* = 15) and control group (*n* = 15). The Wechsler Intelligence Scale was used to measure the intelligence quotient (IQ) of the participants (Jamile Benite Palma Lopes et al., 2017). The inclusion criteria (Eid, 2015; Lee et al., 2016; Ahn, 2021) were: 1) adolescents whose IQ ranged from 50 to 70; 2) adolescents who can follow the therapist’s commands; and 3) adolescents who can independently stand and walk. The exclusion criteria (Cowley et al., 2011; Lee et al., 2016; Ahn, 2021) were: 1) any contraindications to exercise; 2) severe intellectual, visual and hearing disability; and 3) any musculoskeletal, neurological, cardiovascular or respiratory system disorders. This study was approved by the ethics committee of Shandong Sport University. All the parents were requested to sign a written informed consent statement before the study started. The study was registered in the Chinese Clinical Trial Registry (ChiCTR1900021352).

### 2.3 Exercise intervention

The frequency and duration of VR training were designed to be consistent with the educational program outline of the school. The participants in VR group received the 40-min VR training session 3 times a week for 8 weeks. Each session included a 5-min warm-up, 30-min intervention training and 5-min cooldown. The warm-up included 5-min of walking and stretching. The intervention training included the VR training. The cooldown included the stretching exercises. The participants were required to attend at least 24 sessions for the VR training.

**FIGURE 2:**
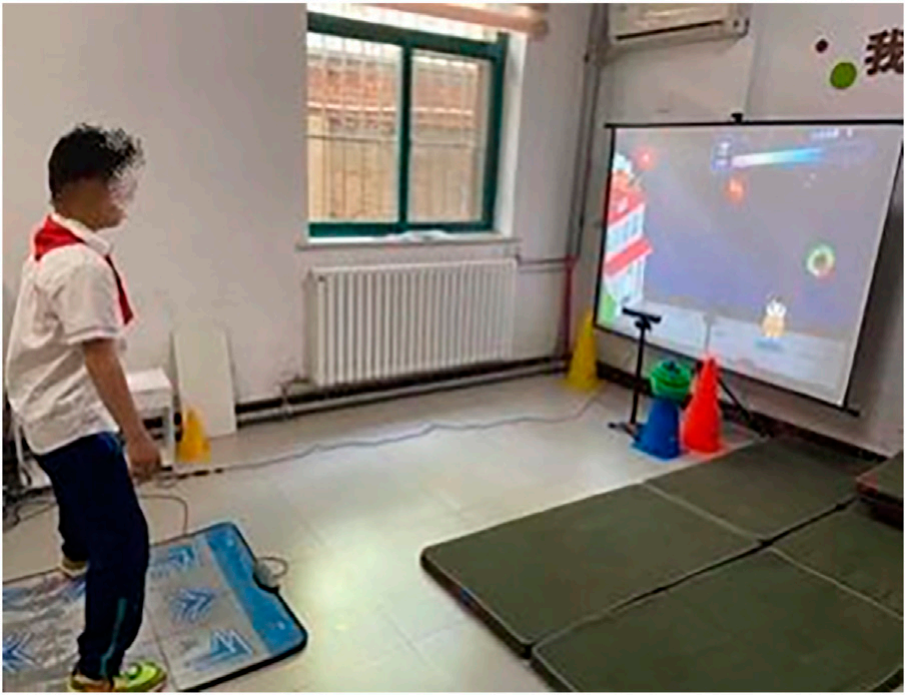
The virtual reality training system.

The participants in the VR group were trained with a VR training system (Xbox Kinect, Shanghai), which included Kinect, console, dance carpet, white screen and projector (Figure 2). The Xbox Kinect, console and monitor were installed in a quiet room. The participants were instructed to remain standing at a distance of 1.5–3.5 m in front of the VR training system. Before the training started, the therapist showed the participants how to use the system. In each session, all the participants played five sports games that the therapist selected in advance among 15 sports according to enjoyment level. The five video games included cool-running, whack a mole, catching gift, racing and playing football, which were designed to enhance the muscle strength, endurance, balance and coordination of adolescents with ID. Each game lasted for 6 min. The participants were individually taught to play the VR training games by the therapist in the first six sessions. After the participants learned how to play the training games, they independently practiced VR training games in the rest of the sessions.

The participants in the control group received the 40-min PE course session 3 times a week for 8 weeks. The PE course included 5-min of warm-up, 30-min of exercise (playing activities, jogging and jumping rope) (Gupta et al., 2011), and 5-min of cooldown. The first six PE course were leaded by a PE teacher who assisted and guided the participants during activities and ensured safety. After that, the participants exercised independently in the PE course during the rest of the sessions.

### 2.4 Outcomes

#### 2.4.1 Balance control

The Berg Balance Scale (BBS) and Timed Up and Go (TUG) test were the most common clinical tests to measure the balance control. These measurements were conducted in a quiet testing room (Juras et al., 2019).

BBS is one of the most commonly used scales in the clinical practice (Sibley. et al., 2011). It is a reliable and standardized tool for balance. It is reported that BBS reliable with the inter-rater reliability estimated at 0.97 (95% CI 0.96–0.98) and intra-rater reliability estimated at 0.98 (95% CI 0.97–0.99) (Downs, 2015). BBS included 14 task items: reaching forward with an outstretched arm, standing with eyes closed with one foot in front, turning, retrieving an object from the floor, standing on one leg, sitting to stand, turning 360°, standing, placing the alternate foot on a stool, transferring, standing with feet together and standing to sitting unsupported (Ekiz et al., 2015). The rating score for each task ranged from 0 to 4 (0 = unable to perform the task; 4 = task is performed independently) (Muir-Hunter et al., 2015), with a maximum score of 56. A greater score in BBS indicated better balance (Downs, 2015).

TUG is a modified test for functional walking. It is a simple method to investigate the dynamic balance. It had a high test-retest reliability (ICC = 0.932) (Martin et al., 2017). The participants stood up from a chair, walked a length of 3 m, turned, walked back and sat on the chair. The time between when the researcher said “go” and when the participant’s back touched the backrest of the chair was recorded in seconds. The total time was recorded with a stopwatch. The test was repeated three times, and the fastest time was recorded. A less time in completing the TUG test indicated better dynamic balance control.

#### 2.4.2 Lower extremity muscle strength

The dominant lower extremity was defined as the preferred leg for kicking a football (Gribble. et al., 2007). The muscle strength of hip flexors, hip extensors, hip abductors, knee flexors, knee extensors and ankle plantar flexors of the dominant leg was measured using a hand-held dynamometer (HHD) (MicroFET3, United States) (Gupta et al., 2011; Wuang et al., 2013) (Table 1). The unit of muscle strength was Newton (N). The ICC values of HHD were 0.95 (hip flexors), 0.84 (hip extensors), 0.96 (knee flexors), 0.93 (knee extensors) and 0.69 (ankle plantar flexors) (Wuang et al., 2013). The researcher held the HDD stationary while the participant applied a maximum force against the dynamometer. Participants were asked to perform test with the maximum force 2–3 s and to maintain until the researcher said to relax. The participants had 30 s of rest between the consecutive trials. Three successful trials were collected for each subject. The collected data were averaged for data analysis.

**TABLE 1:** Muscle strength testing protocol.

Muscle group	Participant position	Dynamometer position
Hip flexors	Sitting, hip flexed at 30°off surface	Resistance given anteriorly to distal thigh
Hip extensors	Prone, knee and thigh extended off surface, pelvis stabilized	Resistance given to posterior distal thigh
Hip abductors	Lateral, hip extented at 20°	Distal fibula
Knee flexors	Sitting, knee flexed at 45°	Heel
Knee extensors	Sitting, knee flexed at 90°	Anterior proximal to lateral malleolus
Ankle plantarflexors	Supine, knee flexed at 90°	Across metatarsal heads

### 2.5 Statistical analysis

The data of participants who attended all three evaluation sessions were included for the final data analysis. All the data were presented using mean ± standard deviation. All data were tested for normality using the Shapiro-Wilk test. Two-way analysis of variance (ANOVA) with repeated-measures was used to compare the outcome data between groups before and after training. If an interaction effect was found, the follow-up tests with Bonferroni adjustments were performed. SPSS 25.0 (SPSS Inc., IL, United States) was used for statistical analysis. A *p*-value less than 0.05 indicated significant difference.

## 3 Results

### 3.1 Baseline characteristics of the participants

A total of 30 participants (15 in the VR group, and 15 in the control group) completed the entire 8-week study without loss of participants. No significant differences were found between the two groups in age, height and body weight (Table 2).

**TABLE 2:** The baseline characteristics of the participants.

	VR group (n = 15)	Control group (n = 15)	*p*
N (male/female)	15 (5/10)	15 (6/9)	0.705
Age (years)	13 ± 0.8	14 ± 2.2	0.087
Height (cm)	1.67 ± 0.11	1.60 ± 0.10	0.069
Weight (kg)	65.46 ± 20.90	61.73 ± 10.26	0.541

Note: VR = virtual reality.

### 3.2 Balance control

Two-way ANOVA with repeated-measures showed no significant interaction effect in BBS (*p* > 0.05) and TUG test (*p* > 0.05) between groups before and after the training (Table 3).

**TABLE 3:** The balance ability in adolescents with ID (X ± S).

	VR group		Control group		*p*		
	Pre-test	Post-test	Pre-test	Post-test	Group	Time	Time × group
BBS (score)	50.66 ± 3.56	52.07 ± 2.94	50.12 ± 4.25	50.62 ± 3.21	0.269	0.269	0.604
TUG (seconds)	9.15 ± 2.01	9.08 ± 2.09	9.42 ± 1.15	9.73 ± 2.01	0.336	0.811	0.700

Note: BBS = berg balance scale; TUG = timed up and go; VR = virtual reality.

### 3.3 Lower extremity muscle strength

Two-way ANOVA with repeated-measures showed a significant interaction effect in the muscle strength of hip extensors (*p* = 0.043), hip abductors (*p* = 0.015) and knee flexors (*p* = 0.006) (Table 4). The follow-up tests showed that muscle strength was significantly improved by 7.3% for hip flexors (*p* < 0.001), 15.3% for hip extensors (*p* = 0.008), 20.4% for hip abductors (*p* = 0.001), 21.6% for knee flexors (*p* = 0.001), 13.9% for knee extensors (*p* < 0.001) and 16.2% for ankle plantar flexors (*p* = 0.001) in the VR group after training compared to the baseline. No significant change in muscle strength was found in the control group after training compared to the baseline (*p* > 0.05). Compared with the control group, significant improvements in the muscle strength were found in hip flexors (*p* < 0.001), hip extensors (*p* = 0.002), hip abductors (*p* < 0.001), knee flexors (*p* < 0.001), knee extensors (*p* = 0.002) and ankle plantar flexors (*p* = 0.042) after the training.

**TABLE 4:** The results of the lower limb muscle strength in adolescents with ID (Newton).

	VR group		Control group		*p*		
	Pre-test	Post-test	Pre-test	Post-test	Group	Time	Time × group
Hip flexors	15.85 ± 1.70	17.00 ± 2.10*^△^	14.29 ± 2.13	14.27 ± 1.87	<0.001	0.258	0.249
Hip extensors	12.03 ± 1.85	13.87 ± 1.21*^△^	11.80 ± 2.11	11.68 ± 2.03	0.075	0.013	0.043
Hip abductors	11.99 ± 2.11	14.43 ± 1.72*^△^	11.58 ± 1.80	11.57 ± 1.83	0.010	0.015	0.015
Knee extensors	14.06 ± 1.51	16.01 ± 1.83*^△^	13.05 ± 1.55	13.70 ± 1.44	<0.001	0.020	0.114
Knee flexors	14.05 ± 1.66	17.08 ± 1.90*^△^	12.63 ± 1.36	13.39 ± 1.22	<0.001	<0.001	0.006
Ankle plantarflexors	12.06 ± 1.72	14.01 ± 1.25*^△^	11.79 ± 1.73	12.81 ± 1.50	0.076	0.010	0.259

Note: VR = virtual reality. *Denotes a significant difference compared with the pre-test value within each group, *p* < 0.05. ^△^Denotes a significant difference between the VR, and control group, *p* < 0.05.

No significant time×group interaction effects were found on the muscle strength of hip flexors (*p* = 0.249), knee extensors (*p* = 0.114) and ankle plantar flexors (*p* = 0.259). Moreover, the main effect of group was found for hip flexors and knee extensors. The analysis of group main effect showed that the muscle strength of hip flexors (*p* < 0.001) and knee extensors (*p* < 0.001) were significantly greater in the VR group than in the control group. After the training, the significant main effect of time was observed on the knee extensors (*p* = 0.02) and ankle plantar flexors (*p* = 0.01).

## 4 Discussion

The aim of this study was to investigate the effect of 8-week VR training on balance and lower extremity muscle strength in adolescents with ID. The results of the study partially supported the two hypotheses.

The results of present study showed that lower limb muscle strength significantly improved after the 8-week VR training in VR group. However, no significant improvement was found in the control group after training. These results partially support the first hypothesis. This finding was consistent with the results of a previous study, in which lower limb muscle strength improved in children with cerebral palsy compared with the control group after an 8-week VR training program (Cho et al., 2016). Another study also reported regular VR training improved the physical fitness in individuals with ID (Lotan et al., 2009). In addition, Carlos et al. reported that individuals with ID improved performance in the virtual task (Carlos Bandeira de Mello Monteiro et al., 2017). Jamile et al. reported the transcranial direct current stimulation combined with Xbox-Kinect game improved the upper limb movement in individuals with ID (Jamile Benite Palma Lopes et al., 2020). Such findings can be explained by the following reasons. First, VR training involves movements with muscle contractions, which may have the strengthening effects. Second, VR training enhances high-intensity, repetitive and task-oriented training (Lee et al., 2019). Third, VR training exhibits other advantages compared to PE course. For example, VR training has been used in neurological rehabilitation as a novel therapeutic method that combines physical activity with the environment. An enriched environment can stimulate the acquisition of motor skills and partially repair neuronal impairment in people with ID (De Giorgio, 2017). This may also be related to visual stimulation. The visual cortex has become the most widely used system for studying the mechanisms of brain plasticity in adulthood (WIESEL. and HUBEL., 1963). VR training provides continuous sound and visual stimulation during practice, acting as a direct support, which in the long term facilitates motor learning (Silas de Oliveira Damasceno et al., 2022). A previous study (Ahn, 2021) found that VR and computer game-based therapy are effective in enhancing the visual perceptual and motor functions. In this study, participants were provided with visual stimulation by seeing images projected onto a screen, which may enhance the strengthening effect. Fourth, improvements in function induced by VR games are thought to be related to interactive training with a wide range of activities and scenarios involving multiple sensory channels and the creation of exercises (Jamile Benite Palma Lopes et al., 2022). And the interaction provided by VR game devices serves as an interesting, encouraging and safe environment.

It is noteworthy that the balance control of the two groups was not significantly improved after training in this study. These findings did not support the first hypothesis. Similar results were found in previous studies that training did not have the effect on improving the balance control in individuals with cerebral palsy or Parkinson’s disease (Wu et al., 2019; Chen et al., 2020). Another study also showed no significant improvement was found in the TUG test score in the VR group and PE group after 8 weeks of training (Hsu, 2016). None of the exercises in the VR training included the activities with spinning movements or maintaining balance with eyes closed. These activities may challenge the balance control of the participants to improve the balance. Lack of these activities may explain that no significant difference was found in BBS and TUG test after training. In the future, these activities should be included in the VR training programs to improve the balance control of individuals with ID. TUG test is a comprehensive test to assess the functional mobility, which requires muscle strength, proprioception, executive function, attention, coordination and control (Mirelman et al., 2018). In one previous study, TUG was significantly improved after 9-week of VR training in the VR group compared to the control group (Yousefi Babadi and Daneshmandi, 2021). In addition, another study also demonstrated significant improvement in TUG after VR training (Steven Phu et al., 2019). This contradiction to our study may be due to the difference in the duration of intervention, as well as the difference in the participants. Thus, our further study should consider to prolong the training periods for the effect of balance improvement to show up. Lower limb muscle strength is a crucial factor for maintaining static and dynamic balance (Lee et al., 2016). However, the present study found that the significantly improvement in lower limb muscle strength but not balance ability in VR group. Therefore, the improvement in muscle strength may not directly related to the balance improvement. Other factors, such as involvement of central nervous system in balance contorl, should be considered in future study.

In this study, no adverse events were reported during the training sessions. Thus, the training program should be safe for adolescents with ID. All the participants completed all the training sessions without drop-outs, which indicated that this type of training had good compliance for adolescents with ID. People with ID are typically inactive, and thus, their physical functions, such as endurance, balance and strength, are less trained than those of their peers without ID (Enkelaar et al., 2012). The enhancements of muscle strength, balance and physical fitness are socially important for adolescents with ID (Shields et al., 2008).

This study had several limitations. The sample size in this study was small, which limited the generalization of the findings. Thus, future study with a larger sample is necessary. In this study, individuals with Down syndrome were included in the sample and could affect the results because they present specific characteristics in muscle force and postural control. In addition, the reliability of HHD measuring muscle strength can be affected by the position of the test (Lue. et al., 2009). Therefore, we should be careful when measuring muscle strength with HDD to ensure the position of the test. Finally, a more objective measure of balance should be included in future study.

## 5 Conclusion

According to the results of this study, we concluded that the 8-week VR training intervention can improve lower extremity muscle strength, but not balance of adolescents with ID. Adolescents with ID in special education school are recommended to add regular VR training in addition to participating in PE course to improve muscle strength. Further studies should be suggested to prolong intervention duration for improving balance.

## Data Availability

The raw data supporting the conclusion of this article will be made available by the authors, without undue reservation.
